# Paternal microbiota impacts offspring: health risks and reproductive insights

**DOI:** 10.1002/mco2.749

**Published:** 2024-10-13

**Authors:** Junyu Wang, Anren Zhang, Shugang Qin

**Affiliations:** ^1^ Department of Rehabilitation Medicine Shanghai Fourth People's Hospital Affiliated to Tongji University School of Medicine Shanghai China; ^2^ Department of Experimental Research Sichuan Clinical Research Center for Cancer, Sichuan Cancer Hospital & Institute, Sichuan Cancer Center, Affiliated Cancer Hospital of University of Electronic Science and Technology of China Chengdu China

1

In a recent study published in *Nature*, Argaw‐Denboba et al. explored the impact of paternal gut microbiota on the health of offspring.^1^ Perturbations in paternal gut microbiota notably impacted offspring health, causing weight issues, developmental disorders, and raised early mortality. Researchers linked these effects to “gut‐germline axis” dysregulation and placental dysfunction, offering new views on preventing adverse pregnancy outcomes (Figure 1).

**FIGURE 1:**
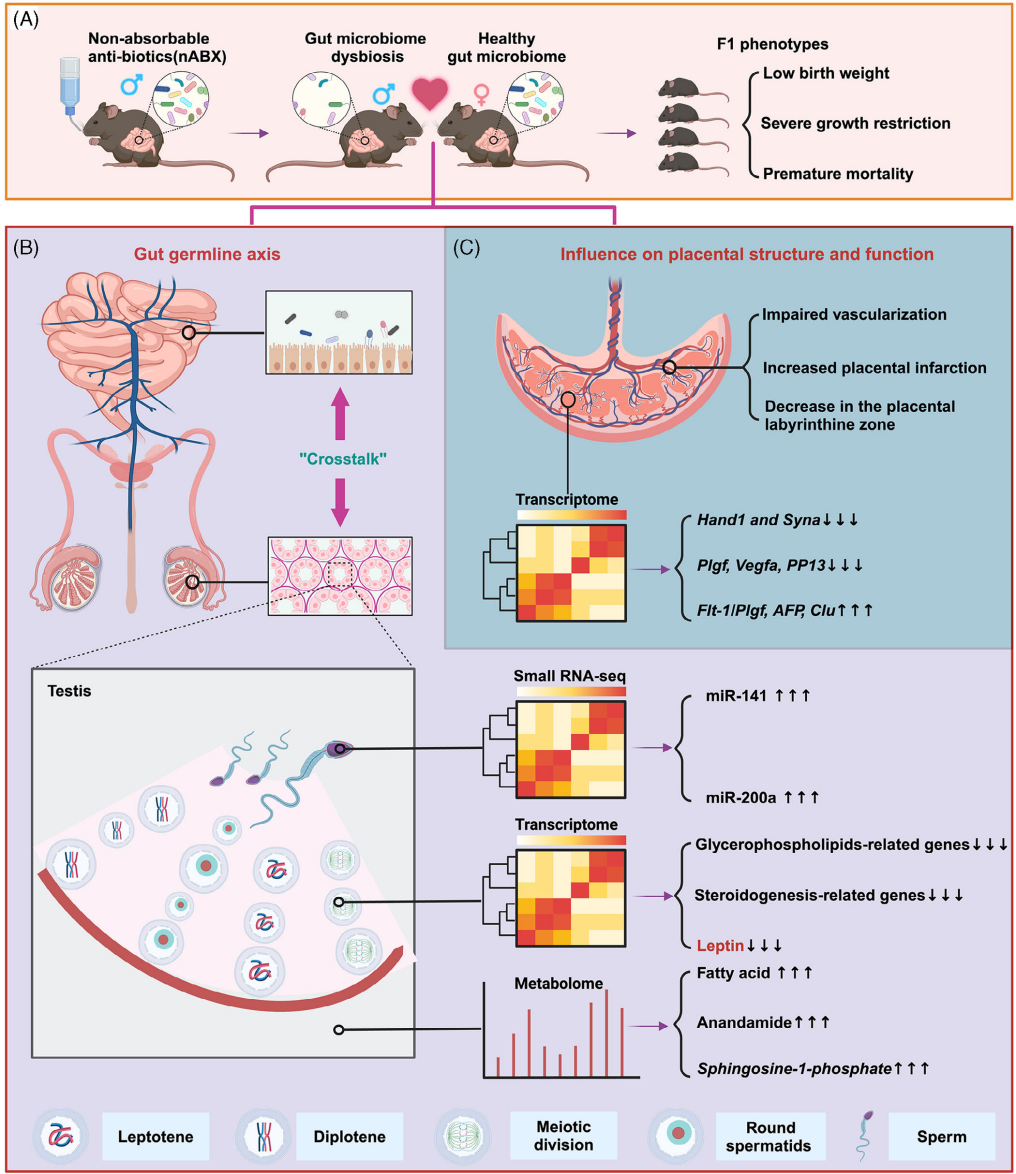
Displays the effects of non‐absorbable antibiotics (nABX)‐induced gut microbiota dysbiosis in paternal mice. Panel (A) shows reduced body weight, growth restriction, and increased early mortality in F1 offspring. Panel (B) illustrates the impact on testicular structure and function, including germ cell loss and reduced sperm count, with increases in fatty acids, cannabinoids, and S1P in testicular tissue. Genes related to leptin and steroidogenesis were downregulated, highlighting leptin's role in the “Gut‐germline axis.” Elevated levels of miR‐141 and miR‐200a were found in sperm. Panel (C) depicts a disrupted placental structure, with reduced labyrinth zone and increased placental infarction, alongside significant changes in clinical markers related to placental insufficiency.

The gut microbiota plays a crucial role in regulating human metabolism, hormone secretion, and immune function, helping to maintain overall physiological health. Recent research has established a connection between gut microbiota and male reproductive health. The study revealed that gut microbiota influences vitamin A metabolism, and any disruptions in this process can affect testicular cells through the bloodstream, ultimately impairing sperm function.^2^ The concept of the “Gut‐testis axis” suggests that gut microbiota can impact interactions between the gut and testes via metabolites, highlighting the complex communication involved. Traditionally, research has primarily focused on the influence of maternal microbiota on fetal development. It was commonly believed that fetal gut microbiota colonization begins during delivery, influenced by maternal skin bacteria, vaginal secretions, and feces. This early colonization plays a significant role in shaping the physiological state of the offspring.^3^ However, the effects of paternal gut microbiota disorders on offspring and the underlying biological mechanisms remain poorly understood, highlighting the need for further research in this area.

Argaw‐Denboba et al. investigated the impact of paternal gut microbiota disruption on offspring health, specifically focusing on physiological status and viability. The research team initiated their study by establishing a male mouse model with disrupted gut microbiota using non‐absorbable antibiotics (nABX). The findings indicated that the offspring of nABX‐treated fathers displayed significantly reduced body weight (*p *= 0.023, nested unpaired t‐test; Control (CON) *n* = 172 (26 L), nABX *n* = 181) (28 L), severe growth restrictions (SGR; body‐weight Z‐score < −3), and a marked increase in early postnatal mortality (*p *= 0.0002, Mantel‐Cox test). To confirm these results, the team employed two additional intervention methods: a combination of antibiotics (avaABX) and an osmotic laxative (polyethylene glycol), which similarly disrupted the paternal gut microbiota. These interventions supported the hypothesis that paternal gut microbiota disruption heightens the risk of developmental disorders and early mortality in offspring.

Further investigations assessed whether reversing the disrupted paternal gut microbiota could mitigate the adverse effects observed in F1 offspring. Upon discontinuing the antibiotics, the researchers noted a reversal in the abnormal physiological characteristics and survival rates of the offspring. Intergenerational transmission experiments then ruled out the transfer of disrupted microbiota from fathers to mothers and F1 offspring, confirming that the observed phenotypes in F1 offspring were not caused by direct microbiota transfer. Finally, through in vitro fertilization experiments, the team established that the abnormal physiological characteristics and survival rates in F1 offspring were specifically transmitted through paternal gametes.

The study introduced the concept of the “Gut‐germline axis” and highlighted the severe disruption of paternal testicular physiology resulting from an imbalance in gut microbiota. This disruption was characterized by significantly reduced testicular mass and sperm count, the formation of vacuoles due to germ cell loss, and a substantial increase in abnormal seminiferous tubules alongside a significant decrease in epithelial thickness. To analyze the molecular‐level response of the reproductive system to this ecological imbalance, the researchers conducted an untargeted metabolomic analysis on the testes of nABX‐treated fathers. This analysis identified 68 significantly differentially expressed metabolites, including fatty acids, cannabinoids, and sphingosine‐1‐phosphate (*S1P*), which predominantly affect male germ cell function.

Argaw‐Denboba et al. found that genes related to glycerophospholipid metabolism and steroidogenesis were particularly affected, consistent with observed metabolic changes. Leptin, produced by germ cells and crucial for energy balance and reproduction, emerged as a key gene. Using leptin‐deficient mice and enzyme‐linked immunosorbent assays, they demonstrated that reduced leptin levels were closely linked to adverse offspring phenotypes, suggesting its role as a critical signal in the “Gut‐germline axis.” The study also revealed that while DNA methylation in sperm from nABX‐treated males remained stable, significant changes occurred in microRNAs, such as miR‐141 and miR‐200a. These findings indicate that gut microbiota imbalances caused by nABX result in complex molecular changes passed to offspring. Previous research indicated that small non‐coding RNAs (sncRNAs) in paternal sperm mediate interactions between genetic material and environmental factors. Gapp et al. showed that traumatic stress altered various microRNAs (miRNAs) in male mice's sperm, affecting offspring behavior and metabolism.^4^ Rodgers et al. found nine miRNAs altered under paternal stress, and their injection into zygotes reduced maternal mRNA reserves in early embryos, reprogramming gene expression in the offspring's hypothalamus.^5^ This reprogramming led to stress‐related phenotypes in offspring, underscoring the importance of sperm miRNAs in transgenerational inheritance and highlighting the need for further investigation into these mechanisms.

The researchers investigated the effects of nABX‐treated fathers on placental structure and function. They found significant disruptions, including reduced labyrinthine zone (*p *= 0.0098), altered vascular structures (*p* = 0.0076), and increased placental infarction (*p *= 0.0296). Key genes involved in placental development, such as Hand1 and Syna, were significantly downregulated, indicating severe impairment. Additionally, markers associated with human placental insufficiency, such as *PlGF*, *VEGF‐A*, and *PP13*, were significantly reduced, while *Flt‐1/PlGF*, *AFP*, and *CLU* were significantly elevated.

In summary, this study highlights the significant impact of paternal gut microbiota dysbiosis on offspring health, causing issues like abnormal weight, developmental delays, and premature death. Notably, restoring the father's gut microbiome reversed these effects in later generations, suggesting a potential treatment. Using animal models, researchers identified changes in the testicular environment, challenging traditional views and underscoring the importance of paternal gut microbiota. These findings expand our understanding of paternal effects on offspring health and suggest new research avenues. However, further studies, especially in primates and humans, are needed to confirm these results and understand the “Gut‐germline axis” in mammals.

## AUTHOR CONTRIBUTIONS

Junyu Wang and Shugang Qin wrote the manuscript and made the figure. Anren Zhang conducted the supervision and revised the manuscript. All authors have read and approved the article.

## CONFLICT OF INTEREST STATEMENT

The authors declare no conflict of interest.

## ETHICS STATEMENT

Not applicable.

## Data Availability

Not applicable.

## References

[1] Argaw‐Denboba A , Schmidt TSB , Di Giacomo M , et al. Paternal microbiome perturbations impact offspring fitness. Nature. 2024;629(8012):652‐659. 38693261 10.1038/s41586-024-07336-w PMC11096121

[2] Zhang T , Sun P , Geng Q , et al. Disrupted spermatogenesis in a metabolic syndrome model: the role of vitamin A metabolism in the gut‐testis axis. Gut. 2022;71(1):78‐87. 33504491 10.1136/gutjnl-2020-323347 PMC8666830

[3] Kennedy KM , Gerlach MJ , Adam T , et al. Fetal meconium does not have a detectable microbiota before birth. Nat Microbiol. 2021;6(7):865‐873. 33972766 10.1038/s41564-021-00904-0

[4] Gapp K , Jawaid A , Sarkies P , et al. Implication of sperm RNAs in transgenerational inheritance of the effects of early trauma in mice. Nat Neurosci. 2014;17(5):667‐669. 24728267 10.1038/nn.3695 PMC4333222

[5] Rodgers AB , Morgan CP , Leu NA , Bale TL . Transgenerational epigenetic programming via sperm microRNA recapitulates effects of paternal stress. Proc Natl Acad Sci U S A. 2015;112(44):13699‐13704. 26483456 10.1073/pnas.1508347112 PMC4640733

